# Lenvatinib as second-line treatment in patients with unresectable hepatocellular carcinoma: A retrospective analysis

**DOI:** 10.3389/fonc.2022.1003426

**Published:** 2022-11-22

**Authors:** He-nan Qin, Zhen Ning, Rui Sun, Chen-xing Jin, Xin Guo, A-man Wang, Ji-wei Liu

**Affiliations:** ^1^ Department of Oncology, The First Affiliated Hospital of Dalian Medical University, Dalian, China; ^2^ Department of General Surgery, The First Affiliated Hospital of Dalian Medical University, Dalian, China

**Keywords:** lenvatinib, hepatocellular carcinoma, second-line treatment, efficacy, safety

## Abstract

**Objective:**

The purpose of this study is to determine the efficacy and safety of lenvatinib as second-line therapy in Chinese patients with unresectable hepatocellular carcinoma (HCC).

**Methods:**

We performed a retrospective analysis of Chinese patients with unresectable HCC who received second-line treatment of lenvatinib at three institutions from November 2018 to February 2022. Demographic and clinicopathologic characteristics, data on the treatment regimens were obtained from medical records. Tumor response was evaluated every 4-6 weeks by modified Response Evaluation Criteria in Solid Tumors (mRECIST).

**Results:**

In total, 50 patients with unresectable HCC who received second-line treatment of lenvatinib were enrolled in this study. The objective response rate (ORR) was 18.0% and the disease control rate (DCR) was 74.0%, respectively. The duration of response (DoR) was 6.0 months. The median progression-free survival (PFS) and overall survival (OS) were 5.0 and 8.5 months, respectively. Patients who received ICIs combined with anti-angiogenic inhibitors as first-line therapy, achieving CR/PR at first-line therapy, with PFS≥6months at first-line therapy had a higher DCR. Univariate and multivariate analysis showed that AFP (ng/ml)<400, absence of extrahepatic metastasis, Child-Pugh A, tumor number<3, ICIs combined with anti-angiogenic inhibitors as first-line therapy, CR/PR to first-line therapy, and PFS≥6months at first-line therapy were independent factors of favorable PFS. Univariate analysis showed that absence of extrahepatic metastasis, tumor number<3, ICIs combined with anti-angiogenic inhibitors as first-line therapy, and PFS≥6months at first-line therapy were significantly associated with longer OS. Multivariate analysis showed that absence of extrahepatic metastasis, Child-Pugh A, tumor number<3, CR/PR to first-line therapy and PFS≥6months at first-line therapy were independent prognostic factors of OS. The majority of AEs were grade 1-2, and were reversible. Grade 3/4 AEs occurred in 12 patients (24.0%) and were mostly connected with hand-foot skin reactions (10.0%), and 10 patients had lenvatinib dose reductions. Two toxicity-related treatment interruptions were attributed to grade 3 hand-foot skin reaction, and grade 4 proteinuria, respectively.

**Conclusion:**

This study confirms the efficacy and safety of lenvatinib as second-line therapy after progression on sorafenib or ICIs combined with anti-angiogenic inhibitors.

## Introduction

Hepatocellular carcinoma (HCC) is the third leading cause of cancer-related deaths worldwide (1). Over half of all HCC patients globally are from China, where the prognosis is extremely poor, with a 5-year survival rate of only 12.1% (2). The liver is the body’s major immune organ, and its anatomical structure and physiological functions contribute to chemoresistance and poor prognosis of HCC (3). Until 2007, there were no effective treatment options for patients with unresectable HCC. Systemic treatment, especially with conventional cytotoxic drugs, is usually ineffective. Sorafenib was the first and only systemic drug approved by the Food and Drug Administration (FDA) as standard treatment for advanced HCC between 2007 and 2016. However, since more than 80% of HCC patients in China have hepatitis B virus (HBV) infection, the survival benefits imparted by sorafenib are limited in comparison to HCC patients in Europe and the United States (4, 5).

Lenvatinib is a small molecular inhibitor targeting vascular endothelial growth factor receptors 1-3, fibroblast growth factor receptors 1-4, platelet-derived growth factor receptors, and the RET as well as KIT (6). In the phase III REFLECT study, lenvatinib showed non-inferiority in terms of overall survival (OS) compared with sorafenib in the first-line therapy of unresectable HCC (7). In the subgroup analysis, lenvatinib was superior to sorafenib in Asia-Pacific patients, with substantial improvements in overall survival (OS), progression-free survival (PFS), and time to progression (TTP) (8). Based on the REFLECT results, the FDA approved lenvatinib for the first-line treatment of patients with advanced HCC.

However, the rapid progress of immunotherapy therapies has dramatically changed the treatment landscape for advanced HCC in recent years. Immune checkpoint therapies are now being incorporated into HCC therapies, and their combination with molecular targeted therapy is emerging as a tool to enhance the immune response. In the phase III IMbrave 150 trial, which was published in 2021, atezolizumab plus bevacizumab showed significant OS and PFS benefits compared to sorafenib in patients with advanced HCC (9). This allows for a new shift in the patterns of first-line treatment in advanced HCC.

Overall, the new combination treatment paradigm appears to be promising, and as a result, an increasing number of patients are opting for immunotherapy combined with anti-angiogenic agents as their first-line treatment. Meanwhile, sorafenib is still being used as the first-line treatment for some individuals with advanced HCC in China (10). The efficacy and safety of lenvatinib as a second-line treatment for individuals who did not receive lenvatinib as a first-line treatment are unknown. Furthermore, little is known about the clinical features of advanced HCC patients who may benefit from second-line lenvatinib treatment. This study aimed to investigate the efficacy, safety, and potential beneficiaries of lenvatinib in patients with unresectable HCC who received sorafenib or immune checkpoint inhibitors (ICIs) combined with antiangiogenic inhibitors for first-line therapy.

## Materials and methods

### Patient selection and diagnosis of hepatocellular carcinoma

**Figure 1:**
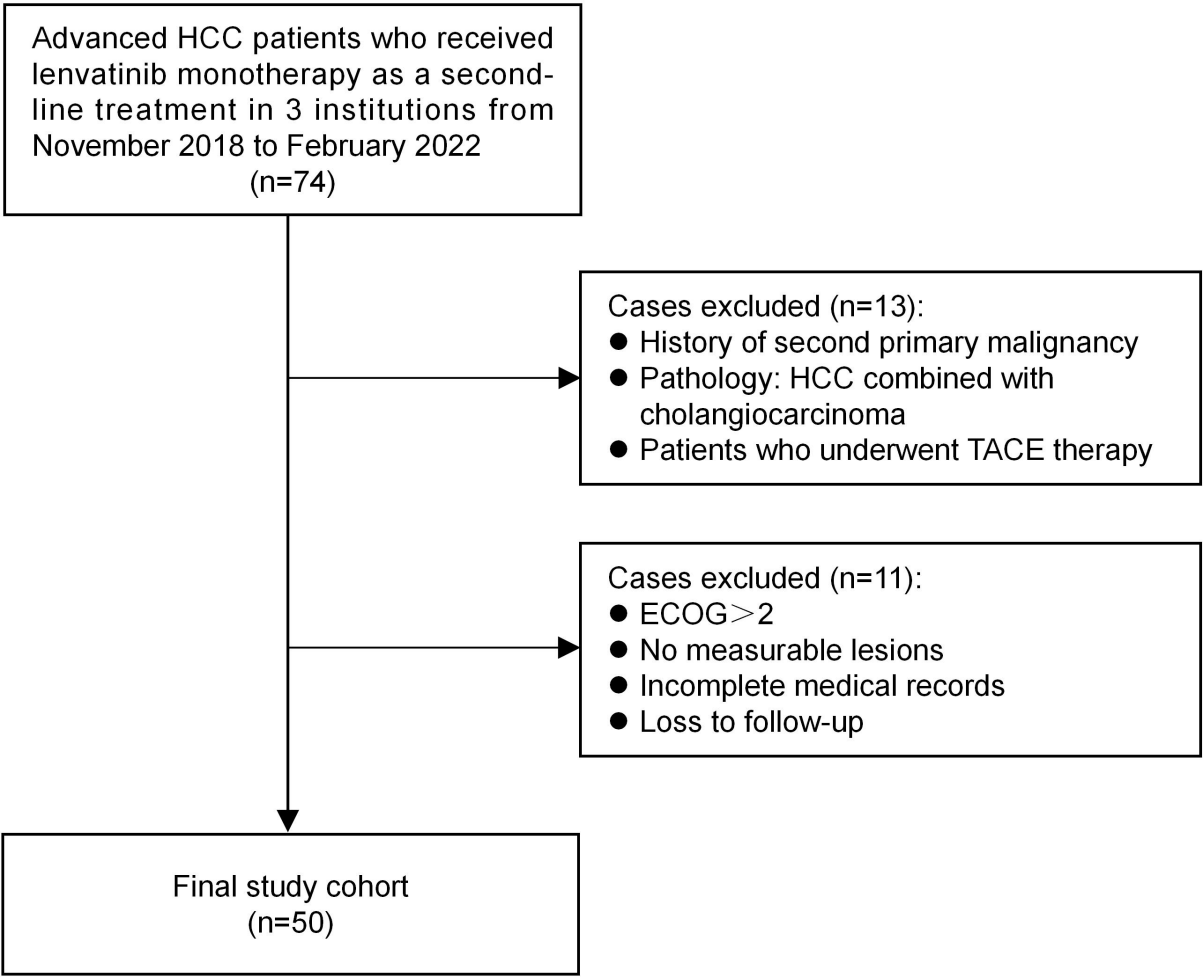
Study flow chart: Flow diagram of patient selection steps.

The study included patients with advanced HCC who received lenvatinib monotherapy as a second-line treatment in 3 institutions (The First Affiliated Hospital of Dalian Medical University, The Second Affiliated Hospital of Dalian Medical University, and Dalian Friendship hospital) from November 2018 to February 2022. The eligible patients must have at least one measurable target lesion for response evaluation, an Eastern Cooperative Oncology Group Performance Status score of 0–2, Barcelona Clinic Liver Cancer Stages (BCLC) B or C categorization, and Child-Pugh class A or B. We excluded patients with a history of second primary malignancy, concurrent cholangiocarcinoma, and patients who underwent TACE therapy. In addition, patients with incomplete clinical records and those lost to follow-up were excluded. 74 patients who received lenvatinib as second-line therapy were screened according to the inclusion and exclusion criteria, and a total of 50 patients were enrolled (**Figure 1**). The diagnosis of HCC was confirmed *via* histology or characteristic radiologic findings, such as dynamic computed tomography (CT) or magnetic resonance imaging (MRI) of the liver. Staging was determined according to the Barcelona Clinic Liver Cancer (BCLC) staging classification at the time of lenvatinib treatment initiation. Alpha-fetoprotein (AFP) was measured at baseline.

### Lenvatinib treatment

The respective standard starting doses of lenvatinib for Child-Pugh A patients weighing 60 kg or more and less than 60 kg were 12 and 8 mg orally once per day. The starting dose of lenvatinib for Child-Pugh class B patients was 8 mg orally once per day[6]. The attending physician decided the dose of lenvatinib according to the grades of adverse events (AEs) or ECOG PS. Treatment was discontinued due to tumor progression, intolerable toxicity, and patient decision.

### Response evaluation and study endpoint

Tumor response was evaluated every 4-6 weeks by modified Response Evaluation Criteria in Solid Tumors (mRECIST) and was assessed as complete response (CR), partial response (PR), stable disease (SD), or progressive disease (PD) (11, 12). The primary endpoint was PFS, defined as the period from the administration of lenvatinib to disease progression or death from any cause. The secondary endpoints were OS, ORR (objective response rate), DCR (disease control rate), and safety. OS is defined as the period from the administration of lenvatinib to death from any cause. ORR is defined as the percentage of patients with a best overall response of CR or PR. DCR is defined as the percentage of patients with the best overall response of CR, PR, or SD. The AEs were evaluated according to the National Cancer Institute Common Terminology Criteria for Adverse Events version 4.0, and the worst grade for each AE was recorded. Bone marrow suppression, liver function, renal function, heart function, and thyroid function were assessed routinely every 2-4 weeks. The patients were followed-up for OS every 30 days until death or study completion. The final follow-up was scheduled for April 2022. The protocol used in this study was approved by the Institutional Ethics Committee, IRB No. PJ-KS-KY-2020-112(X).

### Statistical analysis

The chi-squared test was used to compare the differences between the various patient groups. The Kaplan-Meier method was used to assess the PFS and OS. The hazard ratio (HR) and confidence intervals (CI) were calculated. Univariate analysis was allowed to enter into a multivariable Cox proportional hazards model at a p-value <0.10. A P-value of <0.05 was considered significant. The statistical software used to perform the analyses was SPSS 22.0 and GraphPad Prism 7.0.

## Results

### Baseline characteristics of patients with lenvatinib as second-line treatment

A total of 50 patients with advanced HCC who were treated with lenvatinib as a second-line treatment were enrolled in this study. Baseline patient characteristics are shown in **Table 1**. The majority of patients (62.0%) were male, and the median age was 63.0 years (44–88). Regarding viral hepatitis, 29 patients (58.0%) had HBV infection, 5 patients (10.0%) had hepatitis C virus (HCV) infection, and 16 patients (32.0%) were negative for HBV or HCV, including 10 patients (20.0%) with hepatocirrhosis. 32 patients (64.0%) had an ECOG PS score of 0-1, and 18 patients (36.0%) had a score of 2. The majority of patients (36 patients, 72.0%) had an AFP level of >400ng/ml at baseline, and 24 patients (28.0%) had an AFP response after the initiation of lenvatinib (>20% decrease in AFP serum level from baseline after 4 weeks of lenvatinib treatment). The Child-Pugh classification was A in 27 patients (54.0%) and B in 23 patients (46.0%), whereas the BCLC stage was C in all patients. 34 patients (68.0%) had portal vein tumor thrombus (PVTT), and 35 patients (70.0%) had extrahepatic spread. In the first-line treatment, 20 patients (40.0%) received ICIs combined antiangiogenic inhibitors (atezolizumab plus bevacizumab, 7 patients; sorafenib plus pembrolizumab, 3 patients; apatinib plus camrelizumab, 10 patients), and 30 patients (60.0%) received sorafenib. During the first-line treatment, 1 patient (2.0%) had CR, 15 patients (30.0%) had PR, and 25 patients (50.0%) had SD, 9 patients (18.0%) had PD, and the 3-month and 6-month PFS rates were 86.0% and 50.0%.

**Table 1:** Clinical characteristics in the 50 patients with unresectable HCC who received lenvatinib as second-line treatment.

Characteristics	All (n=50)	First-line treatment		P value
		Sorafenib (n=30)	ICIs+anti-angiogenic inhibitors (n=20)	
Age (years)				0.774
<65	31 (62.0%)	18 (60.0%)	13 (65.0%)	
≥ 65	19 (38.0%)	12(40.0%)	7 (35.0%)	
Gender				0.387
male	31 (62.0%)	17(56.7%)	14 (70.0%)	
female	19 (38.0%)	13(43.3%)	6 (30.0%)	
ECOG-PS				0.765
0-1	32 (64.0%)	20 (66.7%)	12 (60.0%)	
2	18 (36.0%)	10 (33.3%)	8 (40.0%)	
AFP (ng/ml)				1.000
<400	14 (28.0%)	8 (26.7%)	6 (30.0%)	
≥400	36 (72.0%)	22 (73.3%)	14 (70.0%)	
AFP response				0.779
present	24 (48.0%)	15(50.0%)	9 (45.0%)	
absent	26 (52.0%)	15(50.0%)	11 (55.0%)	
Hepatitis				0.312
negative	16 (32.0%)	12(40.0%)	4 (20.0%)	
HBV	29 (58.0%)	15(50.0%)	14 (70.0%)	
HCV	5 (10.0%)	3(10.0%)	2 (10.0%)	
Hepatocirrhosis				0.494
absent	40 (80.0%)	25(83.3%)	15 (75.0%)	
present	10 (20.0%)	5(16.7%)	5 (25.0%)	
Tumor size				0.567
≤3cm	22 (44.0%)	12(40.0%)	10 (50.0%)	
>3cm	28 (56.0%)	18(60.0%)	10 (50.0%)	
Tumor number				0.237
<3	32 (64.0%)	17(56.7%)	15 (75.0%)	
≥3	18 (32.0%)	13(43.3%)	5 (25.0%)	
PVTT				1.000
absent	16 (32.0%)	10(33.3%)	6 (30.0%)	
present	34 (68.0%)	20(66.7%)	14 (70.0%)	
Extrahepatic metastasis				0.114
absent	15 (30.0%)	6(20.0%)	9 (45.0%)	
present	35 (70.0%)	24(80.0%)	11 (55.0%)	
BCLC				–
B	0 (0.00%)	0	0	
C	50 (100.0%)	30(100.0%)	20(100.0%)	
Child-pugh				0.569
A	27 (54.0%)	15(50.0%)	12 (60.0%)	
B	23 (46.0%)	15(50.0%)	8 (40.0%)	
Response to first-line treatment				0.019
CR+PR	16 (32.0%)	7(23.3%)	9 (45.0%)	
SD	25 (50.0%)	14(46.7%)	11 (55.0%)	
PD	9 (18.0%)	9(30.0%)	0	
PFS of first-line treatment				1.000
≥ 6 months	25 (50.0%)	15(50.0%)	10 (50.0%)	
<6 months	25 (50.0%)	15(50.0%)	10 (50.0%)	

ECOG-PS, Eastern Cooperative Oncology Group performance status; AFP, α-fetoprotein; HBV, Hepatitis B virus; PVTT, Portal vein tumor thrombus; BCLC, Barcelona Clinic Liver Cancer; NLR, Neutrophil to lymphocyte ratio, ICIs, immune checkpoint inhibitors, VEGFR, Vascular Endothelial Growth Factor Receptor; PFS, Progression-free survival.

Up to the date of data cutoff, 38 of 50 patients (76.0%) had discontinued lenvatinib, and all had confirmed radiological progression according to mRECIST. 6 of 38 patients (15.8%) received third-line therapy (regorafenib, 3 patients; PD-1 inhibitors, 2 patients; chemotherapy, 1 patient). 5 of 38 patients (13.2%) received TACE, and the others received best supportive care.

### Efficacy of lenvatinib as second-line treatment

**Figure 2:**
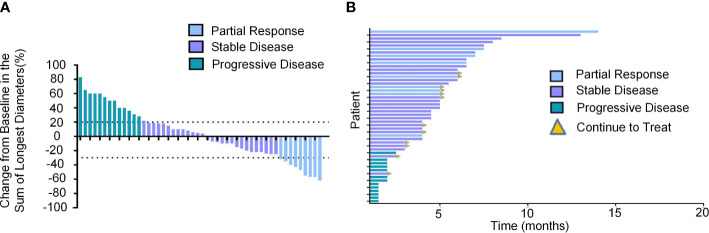
Tumor response of unresectable HCC patients treated with lenvatinib as the second-line treatment. **(A)** Best percentage change from baseline in the sum of the longest diameters of target lesions per response assessment in unresectable HCC patients (n=50). **(B)** DoR in unresectable HCC patients treated with lenvatinib as the second-line treatment.

**Figure 3:**
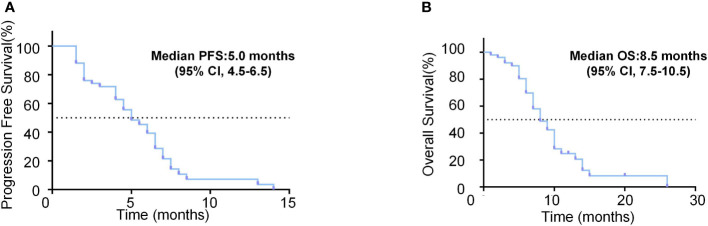
Survival analysis of unresectable HCC patients treated with lenvatinib as the second-line treatment. **(A)** Kaplan-Meier curves of PFS for the unresectable HCC patients treated with lenvatinib as the second-line treatment. **(B)** Kaplan-Meier curves of OS for the unresectable HCC patients treated with lenvatinib as the second-line treatment.

The median observation period after initiation of lenvatinib was 6.3 (5.5-8.0) months. The median treatment duration of lenvatinib was 4.5 months (95% CI, 4.0-5.0). As per mRECIST, no patients had CR, 9 patients (18.0%) had PR, 28 patients (56.0%) had SD, and 13 patients (26.0%) had PD (**Table 2**). The objective response rate (ORR) and disease control rate (DCR) were 18.0% and 74.0% (**Figure 2A**). The duration of response (DoR) was 6.0 months (**Figure 2B**). The median PFS was 5.0 months (95% CI, 4.5-6.5 months, **Figure 3A**). The median OS was 8.5 months (95% CI, 7.5–10.5 months, **Figure 3B**).

**Table 2:** ORR and DCR in the 50 patients with unresectable HCC who received lenvatinib as second-line treatment.

Response to lenvatinib	All (n=50)
CR	0
PR	9
SD	28
PD	13
ORR	18.0%
DCR	74.0%

CR, complete response; PR, partial response; SD, stable disease, or PD, progressive disease; ORR, objective response rate; DCR disease control rate.

### The efficacy of first-line treatment and its effect on second-line lenvatinib

Notably, the modality of first-line therapy may affect the efficacy and outcome of second-line treatment with lenvatinib (**Table 3**). Sorafenib was used as first-line therapy in 30 patients. There were 7 patients with PR to sorafenib previously, and they all achieved SD. Among 14 patients with SD in response to sorafenib, 3 (21.4%) had PR to lenvatinib, and 5 (35.7%) remained SD. Among 9 patients who had PD with sorafenib, none achieved CR/PR to lenvatinib, and 3 (33.3%) had SD. ICIs combined antiangiogenic inhibitors were used as first-line treatment in 20 patients. Among 9 patients with CR/PR in response to ICIs combined antiangiogenic inhibitors, 2(22.2%) had PR to lenvatinib, and 7 (77.8%) remained SD. Among 11 patients with SD in response to ICIs combined antiangiogenic inhibitors, 4 (36.4%) achieved PR to lenvatinib, and 6 (54.5%) had SD.

**Table 3:** The association between the efficacy of first-line treatment and lenvatinib.

Response to lenvatinib	CR+PR	SD	PD	ORR	*P value*	DCR	*P value*
**Response to sorafenib**							
CR+PR (n=7)	0 (0%)	7 (100%)	0 (0%)	0%	0.149	100%	0.025
SD (n=14)	3 (21.4%)	5 (35.7%)	6 (42.9%)	21.4%		57.1%	
PD (n=9)	0 (0%)	3 (33.3%)	6 (66.7%)	0%		33.3%	
**Response to ICIs combined antiangiogenic inhibitors**							
CR+PR (n=9)	2 (22.2%)	7 (77.8%)	0 (0%)	22.2%	0.642	100%	1.000
SD (n=11)	4 (36.4%)	6 (54.5%)	1 (9.1%)	36.4%		90.9%	
PD (n=0)	0(0%)	0(0%)	0(0%)	0%		0%	
**All**							
CR+PR (n=16)	2 (12.5%)	14 (87.5%)	0(0%)	12.5%	0.136	100%	0.001
SD (n=25)	7 (28.0%)	11 (44.0%)	7(28.0%)	28.0%		72.0%	
PD (n=9)	0(0%)	3 (33.3%)	6 (66.7%)	0%		33.3%	
**PFS at first-line therapy**							
PFS≥6months (n=25)	4(16.0%)	17(68.0%)	4(16.0%)	16.0%	0.714	84.0%	0.024
PFS<6months (n=25)	5(20.0%)	10(40.0%)	10(40.0%)	20.0%		60.0%	

CR, complete response; PR, partial response; SD, stable disease, or PD, progressive disease; ORR, objective response rate; DCR disease control rate.

The DCR for lenvatinib second-line therapy was 95.0% in patients who received ICIs combined with anti-angiogenic inhibitors as first-line therapy, which was significantly higher than 60.0% in those who received sorafenib (p=0.006). In patients who achieved CR/PR at first-line therapy, the DCR for lenvatinib second-line therapy was 100.0%, which was significantly higher than those with SD and PD. The efficacy was also significantly different according to the period of progression. In patients with PFS≥6months at first-line therapy, the DCR was 84.0%, which was significantly higher than the 60.0% in those with PFS< 6months (p= 0.024). No statistical difference was observed in ORR.

### Univariate and multivariate analyses of PFS and OS in patients with lenvatinib as second-line treatment

**Figure 4:**
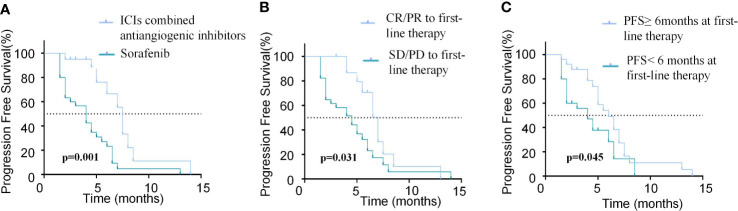
Comparison of PFS in patients with different modalities and efficacy of first-line treatment. **(A)** Kaplan-Meier curves of PFS between patients with ICIs combined antiangiogenic inhibitors or sorafinib for the first-line treatment. **(B)** Kaplan-Meier curves of PFS between patients who achieved CR/PR or SD/PD in the first-line treatment. **(C)** Kaplan-Meier curves of PFS between patients with PFS≥6months or PFS<6months in the first-line treatment.

The results of univariate and multivariate analyses of PFS and OS are listed in **Tables 4**, **5**, respectively. Regarding PFS, univariate analysis showed that AFP (ng/ml)<400 (HR=0.279, 95%CI, 0.102-0.722, p=0.009), absence of extrahepatic metastasis (HR=0.314, 95%CI, 0.136-0.725, p=0.007), Child-Pugh A (HR=0.505, 95%CI, 0.981-0.260, p=0.044), tumor number<3 (HR=0.394, 95%CI, 0.185−0.842, p=0.016), ICIs combined with anti-angiogenic inhibitors as first-line therapy (HR=0.277, 95%CI, 0.131-0.585, p=0.001) (**Figure 4A**), CR/PR to first-line therapy (HR=0.455, 95%CI, 0.222-0.933, p=0.031) (**Figure 4B**) and PFS≥6months at first-line therapy (HR=0.496, 95%CI, 0.251-0.983, p=0.045) (**Figure 4C**) were significantly associated with longer PFS (**Table 4**).

**Table 4:** Univariate and multivariate analysis of PFS in the 50 patients with unresectable HCC who received lenvatinib as second-line treatment.

Factors	Univariate analysis		Multivariate analysis	
	Hazard ratio (95% CI)	*P* value	Hazard ratio (95% CI)	*P* value
Gender (female/male)	0.658 (0.329-1.316)	0.237		
Age (<65 vs. ≥65)	0.788 (0.408-1.522)	0.479		
Hepatitis (absent vs. HBV/HCV)	0.805 (0.402-1.610)	0.538		
Hepatocirrhosis (absent vs. present)	0.787 (0.341-1.815)	0.574		
ECOG-PS (0/1 vs. 2)	0.760 (0.385-1.500)	0.429		
AFP (ng/ml) (<400 vs. ≥400)	0.279 (0.102-0.722)	**0.009**	0.140 (0.042-0.463)	**0.001**
AFP response (ng/ml) (≥20%vs. <20%)	0.637 (0.331-1.227)	0.177		
Extrahepatic metastasis (absent vs. present)	0.314 (0.136-0.725)	**0.007**	0.250 (0.084-0.743)	**0.013**
Child-pugh (A vs. B)	0.505 (0.981-0.260)	**0.044**	0.316 (0.154-0.650)	**0.002**
PVTTs (absent vs. present)	0.880 (0.437-1.771)	0.720		
Tumor size (≤3 vs. >3)	0.911 (0.464-1.789)	0.786		
Tumor number (<3 vs. ≥3)	0.394 (0.185-0.842)	**0.016**	0.337 (0.147-0.776)	**0.011**
First-line treatment (Antiangiogenic inhibitor+ICIs vs. Sorafenib)	0.277 (0.131-0.585)	**0.001**	0.303 (0.107-0.860)	**0.025**
Response to first-line treatment (CR/PR vs. SD/PD)	0.455 (0.222-0.933)	**0.031**	0.308 (0.122-0.772)	**0.012**
PFS of first-line treatment (≥3months vs. <3 months)	0.473 (0.204-1.095)	0.080	0.698 (0.265-1.841)	0.468
PFS of first-line treatment (≥6 months vs. <6 months)	0.496 (0.251-0.983)	**0.045**	0.093 (0.034-0.258)	**0.001**

ECOG-PS, Eastern Cooperative Oncology Group performance status; AFP, α-fetoprotein; HBV, Hepatitis B virus; PVTT, Portal vein tumor thrombus; BCLC, Barcelona Clinic Liver Cancer; NLR, Neutrophil to lymphocyte ratio, ICIs, immune checkpoint inhibitors, VEGFR, Vascular Endothelial Growth Factor Receptor; PFS, Progression-free survival. Bold values, data with p<0.05 in Table.

**Table 5:** Univariate and multivariate analysis of OS in the 50 patients with unresectable HCC who received lenvatinib as second-line treatment.

Factors	Univariate analysis		Multivariate analysis	
	Hazard ratio (95% CI)	P value	Hazard ratio (95% CI)	P value
Gender (female vs.male)	0.635 (0.280-1.437)	0.276		
Age (<65 vs. ≥65)	0.754 (0.365-1.558)	0.446		
Hepatitis (absent vs. HBV/HCV)	0.978 (0.455-2.105)	0.955		
Hepatocirrhosis (absent vs. present)	0.891 (0.339-2.342)	0.815		
ECOG-PS (0/1 vs. 2)	0.959 (0.444-2.072)	0.916		
AFP (ng/ml) (<400 vs. ≥400)	0.306 (0.072-1.295)	0.108		
AFP response (ng/ml) (≥20%vs. <20%)	0.694 (0.333-1.449)	0.331		
Extrahepatic metastasis (absent vs. present)	0.268 (0.081-0.885)	**0.031**	0.196 (0.043-0.884)	**0.034**
Child-pugh (A vs. B)	0.469 (0.217-1.016)	0.055	0.421 (0.184-0.963)	**0.041**
PVTTs (absent vs. present)	0.759 (0.344-1.676)	0.495		
Tumor size (≤3 vs. >3)	0.938 (0.445-1.980)	0.867		
Tumor number (<3 vs. ≥3)	0.320 (0.148-0.694)	**0.004**	0.277 (0.111-0.688)	**0.006**
First-line treatment (Antiangiogenic inhibitor+ICIs-1inhibitor vs. Sorafenib)	0.326 (0.132-0.806)	**0.015**	0.839 (0.260-2-703)	0.769
First-line treatment response (CR/PR vs. SD/PD)	0.460 (0.208-1.019)	0.056	0.206 (0.070-0.605)	**0.004**
PFS of first-line treatment (≥3months vs. <3 months)	0.452 (0.170-1.203)	0.112		
PFS of first-line treatment (≥6 months vs. <6 months)	0.251 (0.113-0.560)	**0.001**	0.147 (0.054-0.397)	**<0.001**

ECOG-PS, Eastern Cooperative Oncology Group performance status; AFP, α-fetoprotein; HBV, Hepatitis B virus; PVTT, Portal vein tumor thrombus; BCLC, Barcelona Clinic Liver Cancer; NLR, Neutrophil to lymphocyte ratio, ICIs, immune checkpoint inhibitors, VEGFR, Vascular Endothelial Growth Factor Receptor; PFS, Progression-free survival. Bold values, data with p<0.05 in Table.

Multivariate analysis revealed that AFP (ng/ml)<400 (HR=0.140, 95%CI, 0.042-0.463, p=0.001), absence of extrahepatic metastasis (HR=0.250, 95%CI, 0.084-0.743, p=0.013), Child-Pugh A (HR=0.316, 95%CI, 0.154-0.650, p=0.002), tumor number<3 (HR=0.337, 95%CI, 0.147-0.776, p=0.011), ICIs combined with anti-angiogenic inhibitors as first-line therapy (HR=0.303, 95%CI, 0.107-0.861, p=0.025), CR/PR to first-line therapy (HR=0.308, 95%CI, 0.122-0.773, p=0.012) and PFS≥6months at first-line therapy (HR=0.093, 95%CI, 0.034-0.258, p=0.001) were independent prognostic factors of favorable PFS (**Table 4**).

**Figure 5:**
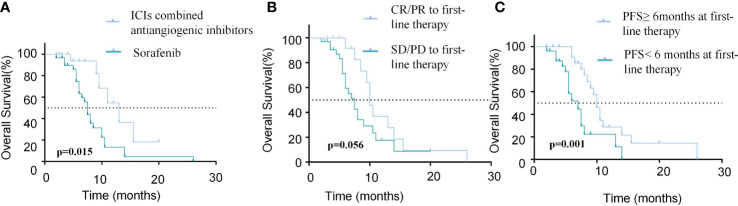
Comparison of OS in patients with different modalities and efficacy of first-line treatment. **(A)** Kaplan-Meier curves of OS between patients with ICIs combined antiangiogenic inhibitors or sorafinib for the first-line treatment. **(B)** Kaplan-Meier curves of OS between patients who achieved CR/PR or SD/PD in the first-line treatment. **(C)** Kaplan-Meier curves of OS between patients with PFS≥6months or PFS<6months in the first-line treatment.

Regarding OS, univariate analysis revealed that absence of extrahepatic metastasis (HR=0.268, 95% CI, 0.081-0.885, p=0.031), tumor number<3 (HR=0.320, 95% CI, 0.148-0.694,p=0.004), ICIs combined with anti-angiogenic inhibitors as first-line therapy (HR=0.326, 95% CI, 0.132-0.806, p=0.015) (**Figure 5A**) and PFS≥6months at first-line therapy (HR=0.251, 95% CI, 0.113-0.560, p <0.001) (**Figure 5C**) were significantly associated with longer OS and CR/PR to first-line therapy (HR=0.460, 95%CI, 0.208-1.019, p=0.056) was not associated with OS (**Figure 5B**). Multivariate analysis showed that absence of extrahepatic metastasis (HR=0.196, 95% CI, 0.043-0.884, p=0.034), Child-Pugh A (HR=0.421, 95%CI, 0.184-0.963, p=0.041), tumor number<3 (HR=0.277, 95% CI, 0.111-0.688,p =0.006), CR/PR to first-line therapy (HR=0.206, 95%CI, 0.070-0.605, p=0.004) and PFS≥6months at first-line therapy (HR=0.147, 95%CI, 0.054-0.397, p<0.001) were independent prognostic factors of OS (**Table 5**).

### Safety of lenvatinib as second-line treatment in patients with unresectable HCC

In total, **Table 6** shows the frequency of adverse events (AEs) after the initiation of lenvatinib treatment in all 50 patients. Treatment-related AEs (TRAEs) were acceptable and no toxicity-related death events occurred. Diarrhea (all grades, 36.0%) and hand-foot skin reaction (all grades, 26.0%) events were the most common toxicities of lenvatinib. The majority of AEs were grade 1-2, and were reversible. Grade 3/4 AEs occurred in 12 patients (24.0%) and were mostly associated with hand-foot skin reactions (10.0%), and 10 patients had lenvatinib dose reductions. Two toxicity-related treatment interruptions were attributed to grade 3 hand-foot skin reaction, and grade 4 proteinuria, respectively. All of the AEs were resolved with the appropriate measures, and most cases were reversible following adequate medical therapy.

**Table 6:** TRAEs of lenvatinib as second-line treatment in patients with unresectable HCC.

TRAEs	All (n=50)		
	Any Grades	Grade 1/2	Grade 3/4
Hand-foot skin reaction	13 (26.0%)	8 (16.0%)	5 (10.0%)
Hypertension	12 (24.0%)	10 (20.0%)	2 (4.0%)
Rash	8 (16.0%)	7 (14.0%)	1 (2.0%)
Fatigue	9 (18.0%)	9 (18.0%)	0 (0.0%)
Hoarseness	4 (8.0%)	4 (8.0%)	0 (0.0%)
Diarrhea	18 (36.0%)	18 (36.0%)	0 (0.0%)
Increased ALT	3 (6.0%)	2 (4.0%)	1 (2.0%)
Increased AST	3 (6.0%)	2 (4.0%)	1 (2.0%)
Increased TB	5 (10.0%)	4 (8.0%)	1 (2.0%)
Decreased WBC	8 (16.0%)	6 (12.0%)	2 (4.0%)
Decreased PLT	2 (4.0%)	2 (4.0%)	0 (0.0%)
Increased creatinine	1 (2.0%)	1 (2.0%)	0 (0.0%)
Proteinuria	1 (2.0%)	0 (0.0%)	1 (2.0%)
Gastrointestinal bleeding	0 (0.0%)	0 (0.0%)	0 (0.0%)

ALT, alanine aminotransferase; AST, aspartate aminotransferase; TB, total bilirubin; WBC, white blood cell; PLT, platelet count.

## Discussion

The purpose of this study was to determine the efficacy and safety of lenvatinib in the second-line setting of unresectable HCC. In our study, patients who received lenvatinib as second-line therapy after sorafenib or ICI in combination with an anti-angiogenic inhibitor had an ORR and DCR of 18.0% and 74.0%, and median PFS and OS of 5.0 months and 8.5 months, respectively. The modalitis of first-line therapy, response to first-line therapy, and PFS of first-line therapy were significantly associated with the outcome of lenvatinib second-line therapy.

In recent years, advances in targeted therapy and immunotherapy have led to an annual increase in treatment options for patients with advanced HCC. The expansion of treatment options complicates systemic HCC treatment, particularly in the selection of second-line treatment options (13). Lenvatinib, a multi-tyrosinase inhibitor with a unique binding mechanism to vascular endothelial growth factor receptor (VEGFR) and fibroblast growth factor receptor (FGFR), has exhibited outstanding antitumor effectiveness and safety in the first-line treatment of unresectable HCC (6). However, clinical data is inadequate to determine if lenvatinib can be utilized as a second-line treatment once sorafenib or ICIs combined with anti-angiogenic therapy fails.

Since 2017, the FDA has granted approval to regorafenib and cabozantinib for advanced HCC following progression on sorafenib. In the phase III RESORCE study, the ORR and DCR in patients who received regorafenib after sorafenib were 10.6% and 65.1%, respectively. Regorafenib was associated with a substantial improvement in PFS (3.1 months vs 1.5 months) and OS (10.6 months vs 7.8 months) when compared to placebo (14). The CELESTIAL study demonstrated a significant survival benefit with cabozantinib in patients previously treated with sorafenib, showing a significant increase versus placebo in PFS (5.2 months vs 1.9 months) and OS (10.2 months vs. 8.0months), while the ORR (4% vs <1%) and DCR (64% vs 33%) were also better in the cabozantinib arm (15, 16). Immune checkpoint inhibitors have been approved as a second-line treatment for advanced HCC in recent years. In the KEYNOTE-224 study, pembrolizumab has shown clinical activity in patients with advanced HCC previously treated with sorafenib. The ORR and DCR were 17% and 62%, respectively, and the median PFS and OS were 4.9 months and 13.2 months. Unfortunately, pembrolizumab failed to show superiority compared to placebo in terms of OS and PFS in the KEYNOTE-240 study (17, 18). The CheckMate 040 study showed that the ORR and DCR of nivolumab for second-line treatment following progression on sorafenib was 15-20% and 58-64%, while the median PFS and OS were 2.1 months and 13.8 months, respectively (19, 20). Camrelizumab, another PD-1 inhibitor, was approved as a second-line therapy in Chinese patients with advanced HCC (21). However, these earlier clinical studies only enrolled patients who had failed to sorafenib in first-line therapy, and did not represent the optimal second-line treatment option in the current HCC treatment landscape. The efficacy of either targeted agents or checkpoint inhibitors in these studies was not very satisfactory. Our study showed a median PFS and OS of 5.0 and 8.5 months for lenvatinib as second-line treatment after progression on sorafenib or ICIs combined with anti-angiogenic inhibitors. But the clinical benefit of second-line lenvatinib treatment needs to be further validated in randomized controlled trials.

In addition, recent studies have evaluated the efficacy of combination therapy modalities in the second-line treatment of advanced HCC, including dual checkpoint inhibitors and ICIs combined with anti-angiogenic agents. In the CheckMate 040 trial, the ORR of nivolumab plus ipilimumab was between 31% and 32%, with DOR ranging between 4.6 and 30.5 months (22). However, combination treatment had more serious AEs compared to the monotherapy arm. Similarly, although the combination of tremelimumab plus durvalumab had an ORR of up to 22.7% and a median OS of up to 18.7 months, it should be noted that the incidence of grade 3 or 4 TRAEs was as high as 35.1%. The phase II RESCUE study showed that the ORR of apatinib in combination with camrelizumab for second-line treatment of advanced HCC was 22.5%, with a median PFS of 5.5 months and a 12-month survival rate of 68.2%. However, the proportion of grade 3 or 4 TRAEs was as high as 77.4% (23, 24). Thus, although the combination treatment modality appears to improve efficacy, the higher incidence of serious adverse effects limits its clinical application in the real world. In our study, lenvatinib showed favorable safety and tolerability.

It is critical to identify the group that will benefit from second-line lenvatinib treatment. Our study revealed that the modality of first-line therapy may affect the efficacy and outcome of second-line treatment with lenvatinib. First-line treatment with ICIs combined with anti-angiogenic agents, CR/PR to first-line therapy, and PFS≥6months at first-line therapy were significantly associated with better DCR, PFS, and OS. Given that anti-angiogenic agents may reprogram the suppressive tumor immune microenvironment by affecting infiltration of immune cells and the expression of immune checkpoints, anti-angiogenic agents combined with ICIs may exert synergistic effects. Previous studies have shown that residual effects persist after discontinuation of ICIs in patients who previously benefited from ICIs, which may explain the better efficacy of second-line treatment with lenvatinib in HCC patients who received ICIs in the first-line treatment (25, 26). In a retrospective analysis, Chen et al. found that response to first-line treatment with sorafenib in patients who failed sorafenib correlated with the efficacy of second-line lenvatinib, which is consistent with the findings of this study, but the study did not analyze the relationship between the PFS of sorafenib first-line therapy and the efficacy of lenvatinib (27). In addition, we found that AFP level, with or without extrahepatic metastasis, Child-Pugh, tumor number, was associated with the efficacy of lenvatinib.

The AEs for lenvatinib in this study were similar to those in the REFLECT study, without unreported AEs (8). Lenvatinib was well tolerated and no treatment-related deaths occurred. The most common adverse events with lenvatinib included diarrhea, hand-foot skin reactions, hypertension, and dermatitis, and most AEs are reversible. This suggests that lenvatinib monotherapy is a relatively well-tolerated treatment option for the second-line HCC population with poor PS scores and more comorbidities.

Our study is a retrospective analysis of real-world data, which includes patients with Child-Pugh B as well as primary PVTT, and presents a more objective overview of the current status of second-line therapy for advanced HCC. Due to the fact that previous trials of second-line treatment for HCC did not enroll patients receiving first-line treatment with ICIs, there is an urgent need to explore the optimal modality for second-line treatment of advanced HCC in the era of immunotherapy. This study confirms the efficacy and safety of lenvatinib as second-line therapy after progression on sorafenib or ICIs combined with anti-angiogenic inhibitors. However, this study has some limitations. First, this was a retrospective analysis with a small sample size, and confounding factors and bias were inevitable. Secondly, some patients received regorafenib, ICIs, or other treatments in third-line therapy after progression on lenvatinib, which may have affected OS outcomes. Thirdly, the AEs of levatinib may have been underestimated due to the nature of retrospective data. Finally, the follow-up period of this study was short, and PFS and OS data need to be updated with further long-term follow-up. Further studies with a larger population or a randomized controlled trial are warranted to validate the findings of this study.

## Data availability statement

The raw data supporting the conclusions of this article will be made available by the authors, without undue reservation.

## Ethics statement

The studies involving human participants were reviewed and approved by Ethics Committee of the First Affiliated Hospital of Dalian Medical University. Written informed consent for participation was not required for this study in accordance with the national legislation and the institutional requirements.

## Author contributions

J-wL and A-mW conceived the project, and J-wL supervised the project. H-nQ and ZN designed and performed most of analysis. RS, C-xJ, and XG provided significant intellectual input. H-nQ, ZN, and A-mW wrote the manuscript with input from all other authors. All authors contributed to the article and approved the submitted version.

## Funding

This study is supported by the National Natural Science Foundation of China grants (No. 82172793, No. 81802272 and No. 81502024).

## Acknowledgments

Thanks to Mohammed Safi for the language modification of this manuscript.

## Conflict of interest

The authors declare that the research was conducted in the absence of any commercial or financial relationships that could be construed as a potential conflict of interest.

## Publisher’s note

All claims expressed in this article are solely those of the authors and do not necessarily represent those of their affiliated organizations, or those of the publisher, the editors and the reviewers. Any product that may be evaluated in this article, or claim that may be made by its manufacturer, is not guaranteed or endorsed by the publisher.

## References

[1] Sung H Ferlay J Siegel RL Laversanne M Soerjomataram I Jemal A. Global cancer statistics 2020: Globocan estimates of incidence and mortality worldwide for 36 cancers in 185 countries. CA Cancer J Clin (2021) 71(3):209–49. doi: 10.3322/caac.21660 33538338

[2] Feng RM Zong YN Cao SM Xu RH. Current cancer situation in China: Good or bad news from the 2018 global cancer statistics? Cancer Commun (Lond) (2019) 39(1):22. doi: 10.1186/s40880-019-0368-6 31030667 PMC6487510

[3] Racanelli V Rehermann B. The liver as an immunological organ. Hepatology (2006) 43(2 Suppl 1):S54–62. doi: 10.1002/hep.21060 16447271

[4] Cheng AL Kang YK Chen Z Tsao CJ Qin S Kim JS. Efficacy and safety of sorafenib in patients in the Asia-pacific region with advanced hepatocellular carcinoma: A phase iii randomised, double-blind, placebo-controlled trial. Lancet Oncol (2009) 10(1):25–34. doi: 10.1016/s1470-2045(08)70285-7 19095497

[5] Llovet JM Ricci S Mazzaferro V Hilgard P Gane E Blanc JF. Sorafenib in advanced hepatocellular carcinoma. N Engl J Med (2008) 359(4):378–90. doi: 10.1056/NEJMoa0708857 18650514

[6] Yamamoto Y Matsui J Matsushima T Obaishi H Miyazaki K Nakamura K. Lenvatinib, an angiogenesis inhibitor targeting Vegfr/Fgfr, shows broad antitumor activity in human tumor xenograft models associated with microvessel density and pericyte coverage. Vasc Cell (2014) 6:18. doi: 10.1186/2045-824x-6-18 25197551 PMC4156793

[7] Kudo M Finn RS Qin S Han KH Ikeda K Piscaglia F. Lenvatinib versus sorafenib in first-line treatment of patients with unresectable hepatocellular carcinoma: A randomised phase 3 non-inferiority trial. Lancet (2018) 391(10126):1163–73. doi: 10.1016/s0140-6736(18)30207-1 29433850

[8] Yamashita T Kudo M Ikeda K Izumi N Tateishi R Ikeda M. Reflect-a phase 3 trial comparing efficacy and safety of lenvatinib to sorafenib for the treatment of unresectable hepatocellular carcinoma: An analysis of Japanese subset. J Gastroenterol (2020) 55(1):113–22. doi: 10.1007/s00535-019-01642-1 PMC6942573 31720835

[9] Galle PR Finn RS Qin S Ikeda M Zhu AX Kim TY. Patient-reported outcomes with atezolizumab plus bevacizumab versus sorafenib in patients with unresectable hepatocellular carcinoma (Imbrave150): An open-label, randomised, phase 3 trial. Lancet Oncol (2021) 22(7):991–1001. doi: 10.1016/s1470-2045(21)00151-0 34051880

[10] Gordan JD Kennedy EB Abou-Alfa GK Beg MS Brower ST Gade TP. Systemic therapy for advanced hepatocellular carcinoma: Asco guideline. J Clin Oncol (2020) 38(36):4317–45. doi: 10.1200/jco.20.02672 33197225

[11] Eisenhauer EA Therasse P Bogaerts J Schwartz LH Sargent D Ford R. New response evaluation criteria in solid tumours: Revised recist guideline (Version 1.1). Eur J Cancer (2009) 45(2):228–47. doi: 10.1016/j.ejca.2008.10.026 19097774

[12] Lencioni R Llovet JM. Modified recist (Mrecist) assessment for hepatocellular carcinoma. Semin Liver Dis (2010) 30(1):52–60. doi: 10.1055/s-0030-1247132 20175033 PMC12268942

[13] Piñero F Silva M Iavarone M. Sequencing of systemic treatment for hepatocellular carcinoma: Second line competitors. World J Gastroenterol (2020) 26(16):1888–900. doi: 10.3748/wjg.v26.i16.1888 PMC7201145 32390700

[14] Bruix J Qin S Merle P Granito A Huang YH Bodoky G. Regorafenib for patients with hepatocellular carcinoma who progressed on sorafenib treatment (Resorce): A randomised, double-blind, placebo-controlled, phase 3 trial. Lancet (2017) 389(10064):56–66. doi: 10.1016/s0140-6736(16)32453-9 27932229

[15] Abou-Alfa GK Meyer T Cheng AL El-Khoueiry AB Rimassa L Ryoo BY. Cabozantinib in patients with advanced and progressing hepatocellular carcinoma. N Engl J Med (2018) 379(1):54–63. doi: 10.1056/NEJMoa1717002 29972759 PMC7523244

[16] Kelley RK Ryoo BY Merle P Park JW Bolondi L Chan SL. Second-line cabozantinib after sorafenib treatment for advanced hepatocellular carcinoma: A subgroup analysis of the phase 3 celestial trial. ESMO Open (2020) 5(4):e000714. doi: 10.1136/esmoopen-2020-000714 32847838 PMC7451459

[17] Zhu AX Finn RS Edeline J Cattan S Ogasawara S Palmer D. Pembrolizumab in patients with advanced hepatocellular carcinoma previously treated with sorafenib (Keynote-224): A non-randomised, open-label phase 2 trial. Lancet Oncol (2018) 19(7):940–52. doi: 10.1016/s1470-2045(18)30351-6 29875066

[18] Kudo M Finn RS Edeline J Cattan S Ogasawara S Palmer DH. Updated efficacy and safety of keynote-224: A phase ii study of pembrolizumab in patients with advanced hepatocellular carcinoma previously treated with sorafenib. Eur J Cancer (2022) 167:1–12. doi: 10.1016/j.ejca.2022.02.009 35364421

[19] El-Khoueiry AB Sangro B Yau T Crocenzi TS Kudo M Hsu C. Nivolumab in patients with advanced hepatocellular carcinoma (Checkmate 040): An open-label, non-comparative, phase 1/2 dose escalation and expansion trial. Lancet (2017) 389(10088):2492–502. doi: 10.1016/s0140-6736(17)31046-2 PMC7539326 28434648

[20] Kudo M Matilla A Santoro A Melero I Gracián AC Acosta-Rivera M. Checkmate 040 cohort 5: A phase I/Ii study of nivolumab in patients with advanced hepatocellular carcinoma and child-pugh b cirrhosis. J Hepatol (2021) 75(3):600–9. doi: 10.1016/j.jhep.2021.04.047 34051329

[21] Qin S Ren Z Meng Z Chen Z Chai X Xiong J. Camrelizumab in patients with previously treated advanced hepatocellular carcinoma: A multicentre, open-label, parallel-group, randomised, phase 2 trial. Lancet Oncol (2020) 21(4):571–80. doi: 10.1016/s1470-2045(20)30011-5 32112738

[22] Yau T Kang YK Kim TY El-Khoueiry AB Santoro A Sangro B. Efficacy and safety of nivolumab plus ipilimumab in patients with advanced hepatocellular carcinoma previously treated with sorafenib: The checkmate 040 randomized clinical trial. JAMA Oncol (2020) 6(11):e204564. doi: 10.1001/jamaoncol.2020.4564 33001135 PMC7530824

[23] Xu J Shen J Gu S Zhang Y Wu L Wu J. Camrelizumab in combination with apatinib in patients with advanced hepatocellular carcinoma (Rescue): A nonrandomized, open-label, phase ii trial. Clin Cancer Res (2021) 27(4):1003–11. doi: 10.1158/1078-0432.Ccr-20-2571 33087333

[24] Mei K Qin S Chen Z Liu Y Wang L Zou J. Camrelizumab in combination with apatinib in second-line or above therapy for advanced primary liver cancer: Cohort a report in a multicenter phase Ib/Ii trial. J Immunother Cancer (2021) 9(3):e002191. doi: 10.1136/jitc-2020-002191 33741732 PMC7986650

[25] Osa A Uenami T Koyama S Fujimoto K Okuzaki D Takimoto T. Clinical implications of monitoring nivolumab immunokinetics in non-small cell lung cancer patients. JCI Insight (2018) 3(19):e59125. doi: 10.1172/jci.insight.59125 30282824 PMC6237460

[26] Yoo C Byeon S Bang Y Cheon J Kim JW Kim JH. Regorafenib in previously treated advanced hepatocellular carcinoma: Impact of prior immunotherapy and adverse events. Liver Int (2020) 40(9):2263–71. doi: 10.1111/liv.14496 32449588

[27] Chen YY Wang CC Liu YW Li WF Chen YH. Clinical impact of lenvatinib in patients with unresectable hepatocellular carcinoma who received sorafenib. PeerJ (2020) 8:e10382. doi: 10.7717/peerj.10382 33240675 PMC7668202

